# 65 Mapping the Literature on PTSD and Depression in Adult Burn Patients: A Scoping Review

**DOI:** 10.1093/jbcr/irae036.057

**Published:** 2024-04-17

**Authors:** Sarah Wang, Brigette Cannata, Medha Vallurupalli, Haig A Yenikomshian, Justin Gillenwater, Sarah A Stoycos

**Affiliations:** Keck School of Medicine of USC, Los Angeles, CA; Keck School of Medicine of USC, Staten Island, NY; Keck School of Medicine, Los Angeles, California; University of Southern California, Los Angeles, CA; Keck Medicine of USC, Los Angeles, CA; Keck School of Medicine of USC, Los Angeles, CA; Keck School of Medicine of USC, Staten Island, NY; Keck School of Medicine, Los Angeles, California; University of Southern California, Los Angeles, CA; Keck Medicine of USC, Los Angeles, CA; Keck School of Medicine of USC, Los Angeles, CA; Keck School of Medicine of USC, Staten Island, NY; Keck School of Medicine, Los Angeles, California; University of Southern California, Los Angeles, CA; Keck Medicine of USC, Los Angeles, CA; Keck School of Medicine of USC, Los Angeles, CA; Keck School of Medicine of USC, Staten Island, NY; Keck School of Medicine, Los Angeles, California; University of Southern California, Los Angeles, CA; Keck Medicine of USC, Los Angeles, CA; Keck School of Medicine of USC, Los Angeles, CA; Keck School of Medicine of USC, Staten Island, NY; Keck School of Medicine, Los Angeles, California; University of Southern California, Los Angeles, CA; Keck Medicine of USC, Los Angeles, CA; Keck School of Medicine of USC, Los Angeles, CA; Keck School of Medicine of USC, Staten Island, NY; Keck School of Medicine, Los Angeles, California; University of Southern California, Los Angeles, CA; Keck Medicine of USC, Los Angeles, CA

## Abstract

**Introduction:**

With nearly 11 million injuries occurring annually worldwide, burns are considered a global health concern. Due to the distressing nature of burns, up to one third of hospitalized burn patients develop post-traumatic stress disorder (PTSD) and up to one half develop clinically significant depressive symptoms, both of which hinder long-term functional recovery. ABA requires mental health screening in verified burn centers, yet a notable gap remains in the identification and standardized screening of psychological issues in burn care and research. The aim of the current study was to conduct a scoping review of the PTSD and depression literature among adult burn patients to provide an overview of the landscape of current research and identify avenues for future research.

**Methods:**

A search was conducted in Pubmed, Embase, and PsychInfo between 1980 - 2023. Inclusion criteria included published in English, adult burn patients (≥ 18), primary sources, and measured PTSD and/or depression after burn. Secondary sources, case reports, scale validation studies, or conference abstracts were excluded. Variables extracted were year published, study design, geographic region of study population, primary and secondary outcomes, and PTSD/depression measurement scales.

**Results:**

The search returned 1073 articles, and after abstract screening 272 articles underwent full text review, with 157 meeting inclusion criteria. Most studies (n = 131, 83%) were published in the year 2000 or later and conducted in high-income countries (n = 133, 85%). PTSD was measured in 117 (75%) articles, depression in 105 (67%), and both in 65 (41%) articles. There was wide variability in measures used to assess PTSD (25 measures) and depression (21 measures). The most frequently used study design was observational, including 48 cross-sectional (31%) and 93 cohort (59%). PTSD and depression after burn injury were often studied in relation with other psychosocial constructs, most commonly pain perception, quality of life, and coping.

**Conclusions:**

Overall, there has been increasing research on PTSD and depression following burn injury in the past two decades. However, they are more often studied as secondary outcomes than as primary outcomes, and intervention research remains nascent. Furthermore, research has overwhelmingly occurred in high-income countries despite the highest incidence of burns occurring in low- and middle-income countries. Areas for future research include evaluating which PTSD and depression screeners are most appropriate for burns; examining PTSD and depression as primary outcomes to understand risk and maintaining factors; and developing effective prevention and intervention programs for PTSD and depression after burn injury.

**Applicability of Research to Practice:**

Our findings can help guide researchers and clinicians in targeting assessments, interventions, and support to improve mental health care for burn survivors.

